# Elevated Prolactin Levels Are Associated With Increased Severity and Psychiatric Symptoms in Anti‐N‐Methyl‐D‐Aspartate Receptor Encephalitis

**DOI:** 10.1002/brb3.70960

**Published:** 2025-10-29

**Authors:** Xiaoyu Ma, Yaxin Lu, Yingying Xu, Shougang Guo, Weiqi Wang, Wei Shang, Wei Qiu, Pin Wang, Yaqing Shu, Yuge Wang

**Affiliations:** ^1^ Department of Neurology The Second Qilu Hospital of Shandong University Jinan China; ^2^ Department of Clinical Data Center The Third Affiliated Hospital of Sun Yat‐Sen University Guangzhou China; ^3^ Department of Neurology Shandong Provincial Hospital Shandong First Medical University Jinan China; ^4^ Department of Neurology The Third Affiliated Hospital of Sun Yat‐Sen University Guangzhou China

**Keywords:** anti‐N‐methyl‐D‐aspartate receptor encephalitis, prolactin, psychiatric symptoms

## Abstract

**Background:**

Autoimmune encephalitis, predominantly anti‐N‐methyl‐D‐aspartate receptor (anti‐NMDAR) encephalitis, is a central nervous system (CNS) autoimmune disorder. Prolactin (PRL), a pro‐inflammatory hormone implicated in autoimmune pathogenesis, may play a role in anti‐NMDAR encephalitis, though its clinical relevance remains unclear.

**Methods:**

Serum PRL levels were compared between anti‐NMDAR encephalitis patients and healthy controls (HCs). Correlations between PRL levels and disease severity were analyzed. Subgroup analyses were performed based on clinical manifestations, tumor comorbidity, ICU admission, disease severity, and MRI abnormalities. Longitudinal PRL levels and modified Rankin Scale (mRS) scores were evaluated in 28 patients during follow‐up. In addition, associations between PRL and inflammatory markers, including neutrophil‐to‐lymphocyte ratio (NLR), were investigated.

**Results:**

Anti‐NMDAR encephalitis patients exhibited significantly elevated PRL levels compared to HCs (*p* < 0.001), with PRL positively correlated with baseline disease severity. After a 3‐month treatment period, both PRL levels and mRS scores decreased significantly (*p* = 0.002, *p* < 0.001, respectively), and their reductions showed a positive correlation (*r* = 0.407, *p* = 0.032). PRL levels were associated with psychiatric symptoms but not with seizures, prodromal fever, speech deficits, or sleep disturbances. Furthermore, PRL abundance correlated with systemic inflammatory markers, notably NLR.

**Conclusions:**

Circulating PRL levels are significantly elevated in anti‐NMDAR encephalitis and correlate with disease activity, suggesting its potential utility as a biomarker for monitoring disease progression. The pathophysiological role of PRL—whether pathogenic or protective—warrants further investigation.

## Graphical abstract

Association of serum PRL with anti‐NMDAR encephalitis.

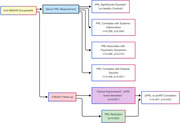

## 1 Introduction

Autoimmune encephalitis (AE) is a central nervous system (CNS) disorder, with anti‐N‐methyl‐D‐aspartate receptor (anti‐NMDAR) encephalitis being the most prevalent subtype (Graus et al. 2016); (Dalmau et al. 2019). This condition manifests with diverse neurological symptoms, including cognitive decline, psychiatric disturbances, seizures, speech impairments, and autonomic dysfunction. The etiology of anti‐NMDAR encephalitis remains poorly understood, although teratomas, post‐herpetic infections, and genetic interactions are believed to be among the potential triggers (Dalmau et al. 2019); (X. Liu, Zheng, et al. 2024). Notably, a striking female predominance (4:1 female‐to‐male ratio) suggests hormonal or sex‐specific immunological factors may contribute to disease susceptibility (Dalmau et al. 2019).

Accumulating evidence highlights the role of sex hormones in modulating immune responses (Orbach et al. 2007). Prolactin (PRL), a pleiotropic polypeptide hormone primarily secreted by anterior pituitary lactotroph cells under hypothalamic dopaminergic inhibition, also exhibits extra‐pituitary synthesis in immune cells (e.g., lymphocytes, monocytes, macrophages), neural tissues, and peripheral organs (Orbach et al. 2007). Within the CNS, PRL actively regulates stress responses by suppressing the hypothalamic–pituitary–adrenal (HPA) axis, crosses the blood–brain barrier (BBB), and influences neurogenesis (Reavley et al. 1997). PRL displays both pro‐ and anti‐inflammatory effects in immune processes (Costanza and Pedotti 2016); (Carranza Lira et al. 2021). Beyond its canonical role in lactation, PRL is implicated in oncogenesis, with prolactin receptor (PRLR) overexpression observed in breast cancer, prostate cancer, hepatocellular carcinoma, and glioblastoma (Alkharusi et al. 2021). Clinically, PRL dysregulation is linked to autoimmune disorders such as systemic lupus erythematosus (SLE), rheumatoid arthritis (RA), and Sjögren's syndrome (Y. Liu et al. 2019). In neurological diseases, elevated PRL levels are reported in Alzheimer's disease (AD), Parkinson's disease (PD), Huntington's disease (HD), multiple sclerosis (MS), cerebral ischemia, and epilepsy (Zverova et al. 2018; Nguyen et al. 2021, Duc Nguyen et al. 2021; Ramos‐Martinez et al. 2021; H. Liu, Zhang, et al. 2024). Mechanistically, PRL modulates local immune microenvironments, activates leukocytes, promotes lymphocyte differentiation, and enhance cytokine production (e.g., TNF‐α, IFN‐γ, IL‐1β) and immunoglobulin synthesis (Tripathi and Sodhi 2008). For instance, it was shown that PRL‐PRLR interactions upregulate TNF‐α expression in peripheral CD14+ monocytes (Tang et al. 2014).

These findings collectively suggest a potential role for PRL in anti‐NMDAR encephalitis pathogenesis. However, PRL involvement in AE remains undefined, and its neuropsychiatric effects appear paradoxical. While PRL may promote neuroprotection via neurogenesis, excessive levels correlate with suppressed neurogenesis and depressive‐like behaviors (Torner 2016). Despite these implications, there has been a lack of studies to date to systematically examine the relationship between circulating PRL levels in anti‐NMDAR encephalitis and their clinical relevance. To address this gap, we conducted a prospective study to investigate serum PRL dynamics in patients of anti‐NMDAR encephalitis and evaluate its potential correlation with disease severity, inflammatory markers, and clinical outcomes.

## 2 Material and Methods

### 2.1 Patients and Controls

In compliance with informed consent guidelines, we studied patients diagnosed with anti‐NMDAR encephalitis in the Third Affiliated Hospital of Sun Yat‐Sen University and The Second Qilu Hospital of Shandong University from March 2014 to May 2024. The diagnosis was based on the criteria proposed by Dalmau's team in 2016 (Graus et al. 2016), with pregnant patients excluded. Clinical manifestations, gender, age, and other epidemiological data were collected, along with cerebrospinal fluid (CSF) routine, blood routine, and MRI results. Among our 85 patients, the use of drugs (e.g., antipsychotics and antidepressants) is inevitable, but such cases were relatively rare. For instance, three patients used olanzapine, two patients used haloperidol, one patient used flupentixol and melitracen, and one patient used quetiapine. Healthy controls (HCs) were defined as subjects devoid of pregnancy, any history of tumor, inflammatory or autoimmune diseases, neurological, or psychiatric disorders. At the same time, the use of various medications (such as antipsychotics, antiemetics, and antidepressants) is also prohibited. The patients received first‐or second‐line therapies. The first‐line therapy, including intravenous methylprednisolone (IVMP), intravenous immunoglobulin (IVIg), and plasma exchange, are widely utilized in patients with AE. All patients experiencing their first episodes received the first‐line immunotherapy. Second‐line immunotherapies, such as anti‐CD20 monoclonal antibodies (e.g., rituximab), mycophenolate mofetil (MMF), azathioprine, tacrolimus, were applied to patients exhibiting suboptimal responses to the first‐line treatment. The neurological status of each patient was assessed using the modified Rankin Scale (mRS), with a score ranging from 0 to 6, as follows: *no symptoms* (0); *no significant disability despite symptoms* (1); *slight disability* (2); *moderate disability* (3); *moderate‐to‐severe disability* (4); *severe disability* (5); *and death* (6). If there is no improvement within 1 month after immunotherapy or tumor resection and mRS score is maintained at 4 or higher, the initial treatment was recorded as failure (limited response).

### 2.2 Ethnics Statement and Consent to Participate

The study was approved by the Research Ethics Committee of The Third Affiliated Hospital of Sun Yat‐Sen University [(2019)2‐637] and The Second Qilu Hospital of Shandong University (KYLL2024899). Informed written consent was obtained from all patients or their representatives.

### 2.3 Determination of the Levels of Six Sex Hormones

Aliquots of at least 2 mL of early morning fasting peripheral blood were collected by nurses in a uniform manner in the early morning and sent to the hospital laboratories for analyses. After 30 min standing, the samples were centrifuged to obtain fresh serum which were then we measured for levels of six sex hormones: follicle‐stimulating hormone (FSH), luteinizing hormone (LH), PRL, estradiol, progesterone, and testosterone.

### 2.4 Follow‐Up Evaluations

Of the 85 patients with anti‐NMDAR encephalitis, 28 were followed up with a repeat assessment of mRS scores and circulating PRL levels at 3 months after admission.

### 2.5 Statistical Analysis

R‐3.6.2 software and SPSS 26.0 software were used for statistical analysis, and GraphPad Prism 9.4 was used to plot graphs. All continuous variables are presented as mean (standard deviation) if the data were normally distributed, or as medians [Q1, Q3] if the data were not normally distributed. The Mann–Whitney *U*‐test was performed to assess the significance of differences between two groups. Logistic regression was used to adjust for the effects of age and gender. Spearman's rank correlation coefficients were used to evaluate correlations between PRL and different clinical parameters. *p* < 0.05 was considered statistically significant.

## 3 Results

### 3.1 Demographic and Clinical Features

As shown in Table 1, 85 patients with anti‐NMDAR encephalitis (median age, 22.0 years; male:female = 28:57) and 152 HCs (median age, 22.5 years; male:female = 50:102) were enrolled to compare the levels of six sex hormones and determine if there exists any correlation with clinical symptoms and inflammatory status of the patients. The median mRS score of the patients was 4. Of the 85 patients, 36 patients (42.35%) had prodromal symptoms (fever, headache), and 48 patients (56.47%) had cognitive disorder, 75 (88.24%) had psychiatric and behavioral abnormalities in this study. Moreover, 26 (30.59%) had speech disorder, 46 (54.12%) had seizures, 16 (18.82%) had movement disturbance, 30 (35.29%) had disturbance of consciousness, 10 (11.76%) had central hypoventilation, and 24 (28.24%) had involuntary movement. 37 (43.53%) patients had an abnormal MRI. In total, 24 (28.24%) had tumors, including ovarian teratoma (17 cases, 20.00%), ovarian cysts (6 cases, 7.06%), and thyroid cancer (1 case, 1.18%). In CSF routine, the median of CSF WBC was 12, and the median of CSF total protein was 0.250. In blood routine, among all the patients, white blood cell (WBC) was 10.74 ± 4.27 × 10^9^, monocytes are 0.76 ± 0.40 × 10^9^, neutrophils are 8.14 ± 4.18 × 10^9^, and lymphocytes are 1.63 ± 0.70 × 10^9^. The median of neutrophil‐to‐lymphocyte ratio (NLR) was 4.87. A total of 30 (35.29%) patients had been admitted to ICU. The median hospital stay was 28 days. In terms of treatment, 42 (49.41%) received first‐line therapy, and 43 (50.59%) received first‐line and second‐line combination therapy.

**TABLE 1:** Demographic features of patients with anti‐NMDAR encephalitis and healthy controls.

Characteristics	Anti‐NMDAR encephalitis (*n* = 85)		Healthy controls (*n* = 152)		Unadjusted *p* value	Adjusted *p* value*
	Male	Female	Male	Female		
**Age** (years, median [Q1,Q3])	21.5 [15.0,32.2]	22.0 [18.0,28.0]	24.5 [16.2,30.8]	22.0 [15.2,31.8]	0.979	—
**Gender**	28	57	50	102	1.000	—
**Testosterone** (nmol/L, mean [SD])	11.43 (6.53)	1.14 (0.64)	17.15 (7.66)	1.21 (0.59)	0.184	0.002
**FSH** (mIU/mL, mean [SD])	5.55 (4.72)	9.96 (18.46)	4.24 (2.07)	10.87 (16.77)	0.017	0.872
**Progesterone** (nmol/L, mean [SD])	1.93 (4.78)	4.92 (13.85)	0.54 (0.45)	4.47 (12.08)	0.010	0.775
**LH** (mIU/mL, mean (SD))	4.40 (3.71)	7.96 (12.17)	3.56 (1.89)	7.24 (8.44)	0.116	0.492
**estradiol** (pmol/L, mean [SD])	99.85 (57.35)	213.77 (302.23)	79.52 (35.00)	249.43 (301.65)	0.815	0.612
**PRL** (mIU/L, mean [SD])	597.48 (511.60)	1006.16 (842.32)	213.19 (77.73)	300.82 (139.52)	< 0.001	< 0.001
**mRS scores** (median [Q1,Q3])	4.00 [3.00,4.00]	4.00 [3.00,4.00]	—	—	—	—
**Clinical symptoms** (*n*, %)						
Prodromal symptoms (fever, headache)	9 (8.23)	27 (31.76)	—		—	—
Cognitive disorder	14 (16.47)	34 (40.00)	—		—	—
Psychiatric and behavioral abnormalities	23 (27.06)	52 (61.18)	—		—	—
Speech disorder	9 (8.23)	17 (20.00)	—		—	—
Seizures	17 (20.00)	29 (34.12)	—		—	—
Movement disturbance	6 (7.06)	10 (11.76)	—		—	—
Disturbance of consciousness	9 (8.23)	21 (24.71)	—		—	—
Central hypoventilation	2 (2.35)	8 (9.41)	—		—	—
Involuntary movement	7 (8.24)	17 (20.00)	—		—	—
**Abnormal MRI** (*n*, %)	11 (12.94)	26 (30.59)	—		—	—
**CSF routine**						
CSF WBC (×106, median [Q1,Q3])	8.50 [2.00;29.0]	14.0 [2.00;42.0]	—		—	—
CSF TP (g/L, median [Q1,Q3])	0.26 [0.20;0.55]	0.24 [0.18;0.33]	—		—	—
**Blood routine**						
WBC (×109, mean (SD))	10.69 (3.84)	10.76((4.51)	—		—	—
Monocyte (×109, mean (SD))	0.73 (0.38)	0.78 (0.41)	—		—	—
Neutrophils (×109, mean (SD))	8.18 (3.66)	8.11 (4.45)	—		—	—
Lymphocytes (×109, mean (SD))	1.67 (0.86)	1.61 (0.60)	—		—	—
NLR (median [Q1,Q3])	4.00 [3.34;9.88]	5.26 [2.66;7.66]			—	—
**Treatment (*n*, %)**						
First‐line treatment	11 (12.94)	31 (36.47)	—		—	—
First‐line combined with second‐line treatment	17 (20.00)	26 (30.59)	—		—	—
**Hospital stay** (days, median [Q1,Q3])	25.5 [19.8;38.0]	28.0 [17.0;40.0]	—		—	—
**ICU support** (*n*, %)	9 (10.59)	21 (24.71)	—		—	—
**Tumors (*n*, %)**						
Ovarian teratoma	0	17 (20.00)	—		—	—
Ovarian cysts	0	6 (7.06)	—		—	—
Thyroid cancer	1 (1.18)	0	—		—	—

Abbreviations: Anti‐NMDAR, anti‐N‐Methyl‐D‐aspartate receptor; CSF, cerebrospinal fluid; FSH, Follicle‐stimulating hormone; LH, luteinizing hormone; mRS, modified Rankin Scale; NLR: neutrophil‐to‐lymphocyte ratio. *p*, anti‐NMDARE (total) *v*. HCs; PRL, prolactin; SD, standard deviation; TP, total protein; WBC, white blood cells. *After adjusting for the effects of age and gender.

### 3.2 Comparison of Six Circulating Sex Hormones Levels Between Patients With Anti‐NMDAR Encephalitis and HCs

**FIGURE 1:**
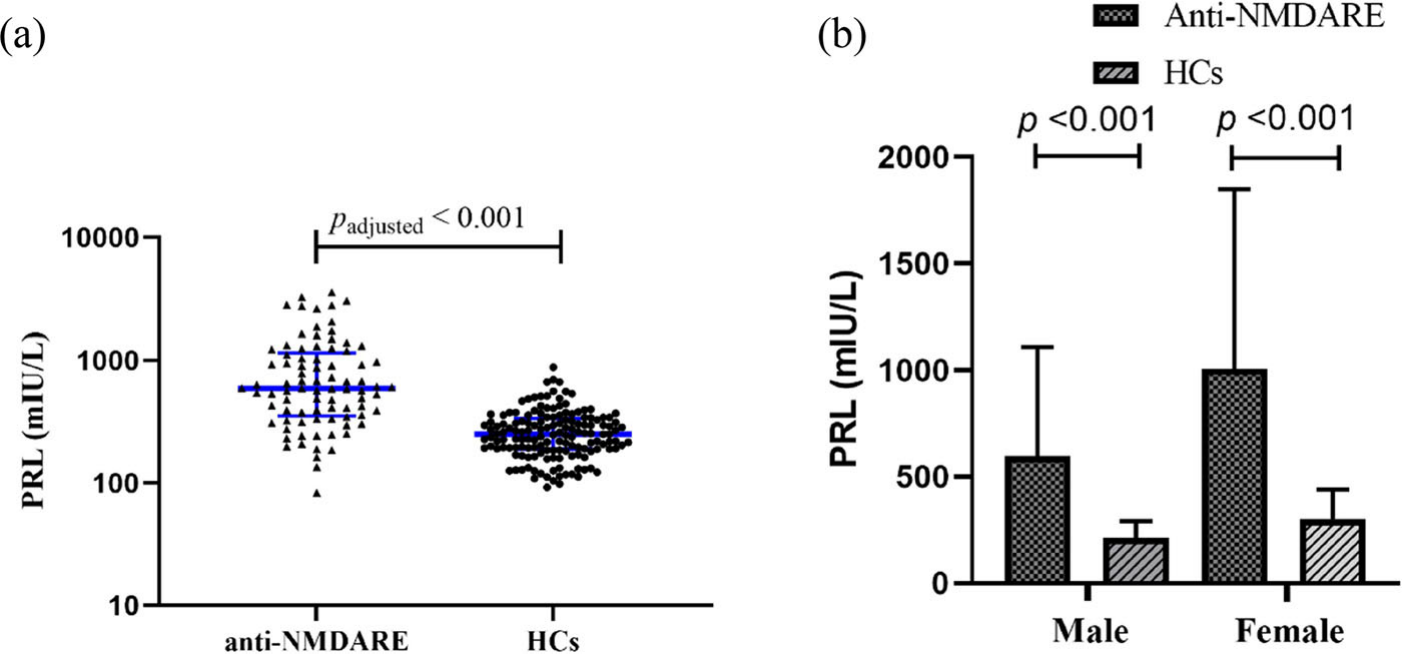
The PRL levels in anti‐NMDAR encephalitis compared with HCs. (a) Compared with HCs (*n* = 152), PRL levels were significantly higher in anti‐NMDAR encephalitis (anti‐NMDARE, *n* = 85) at initial admission (*p* < 0.001, *p*_adjusted_ < 0.001). **(b)** PRL levels were significantly higher in males with anti‐NMDAR encephalitis (*n* = 28) compared with male healthy controls (*n* = 50, *p* < 0.001), and PRL levels were also higher in females with anti‐NMDAR encephalitis (*n* = 57) compared with female healthy controls (*n* = 102, *p* < 0.001).

**FIGURE 2:**
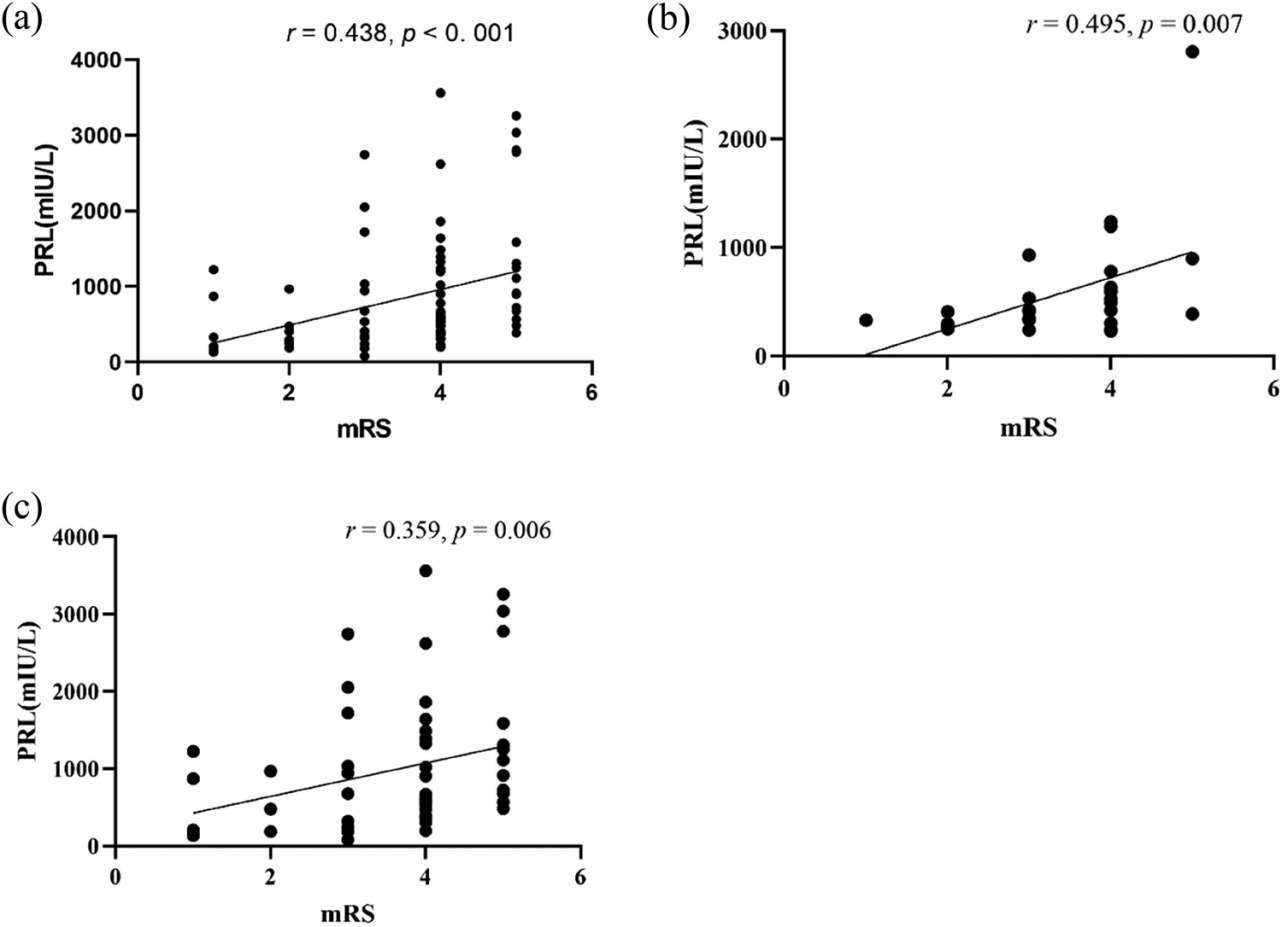
Relationship between PRL and disease severity. (a) In all patients. (b) In male. (c) In female.

We evaluated the levels of six circulating sex hormones levels between patients with anti‐NMDAR encephalitis and HCs. PRL (male: 597.48 ± 511.60 mIU/L, female: 1006.16 ± 842.32 mIU/L vs male: 213.19 ± 77.73 mIU/L, female: 300.82 ± 139.52 mIU/L, *p* < 0.001, *p*_adjusted_ < 0.001) (Table 1 and Figure 1) and testosterone (male: 11.43 ± 6.53 nmol/L, female: 1.14 ± 0.64 nmol/L vs male: 17.15 ± 7.66 nmol/L, female: 1.21 ± 0.59 nmol/L, *p* = 0.184, *p*_adjusted_ = 0.002) (Table 1 and Figure S1) were increased significantly as compared with the control groups. Moreover, male patients had significantly higher serum levels of PRL than male HCs (597.48 ± 511.60 mIU/L vs. 213.19 ± 77.73 mIU/L, *p* < 0.001), female patients also had significantly higher serum levels of PRL than male HCs (1006.16 ± 842.32 mIU/L vs. 300.82 ± 139.52 mIU/L, *p* < 0.001). Male patients had significantly lower serum levels of testosterone than male HCs (11.43 ± 6.53 nmol/L vs. 17.15 ± 7.66 nmol/L, *p* = 0.001). There were no significant differences in other four hormone indicators, including FSH, progesterone, LH, and estradiol, after gender and sex adjustment. We then evaluated the correlation between PRL, testosterone and the disease severity. PRL levels was positively correlated with the mRS score at initial in all anti‐NMDAR encephalitis patients (*r* = 0.438, *p* < 0.001) and in both male and female subgroups (male: *r* = 0.495, *p* = 0.007; female: *r* = 0.359, *p* = 0.006) (Figure 2). There is no significant correlation between testosterone and the mRS score (*r* = −0.085, *p* = 0.441), both in male and female (Figure S2).

### 3.3 Association Between Circulating PRL Levels and Clinical Characteristics in Patients With Anti‐NMDAR Encephalitis

**FIGURE 3:**
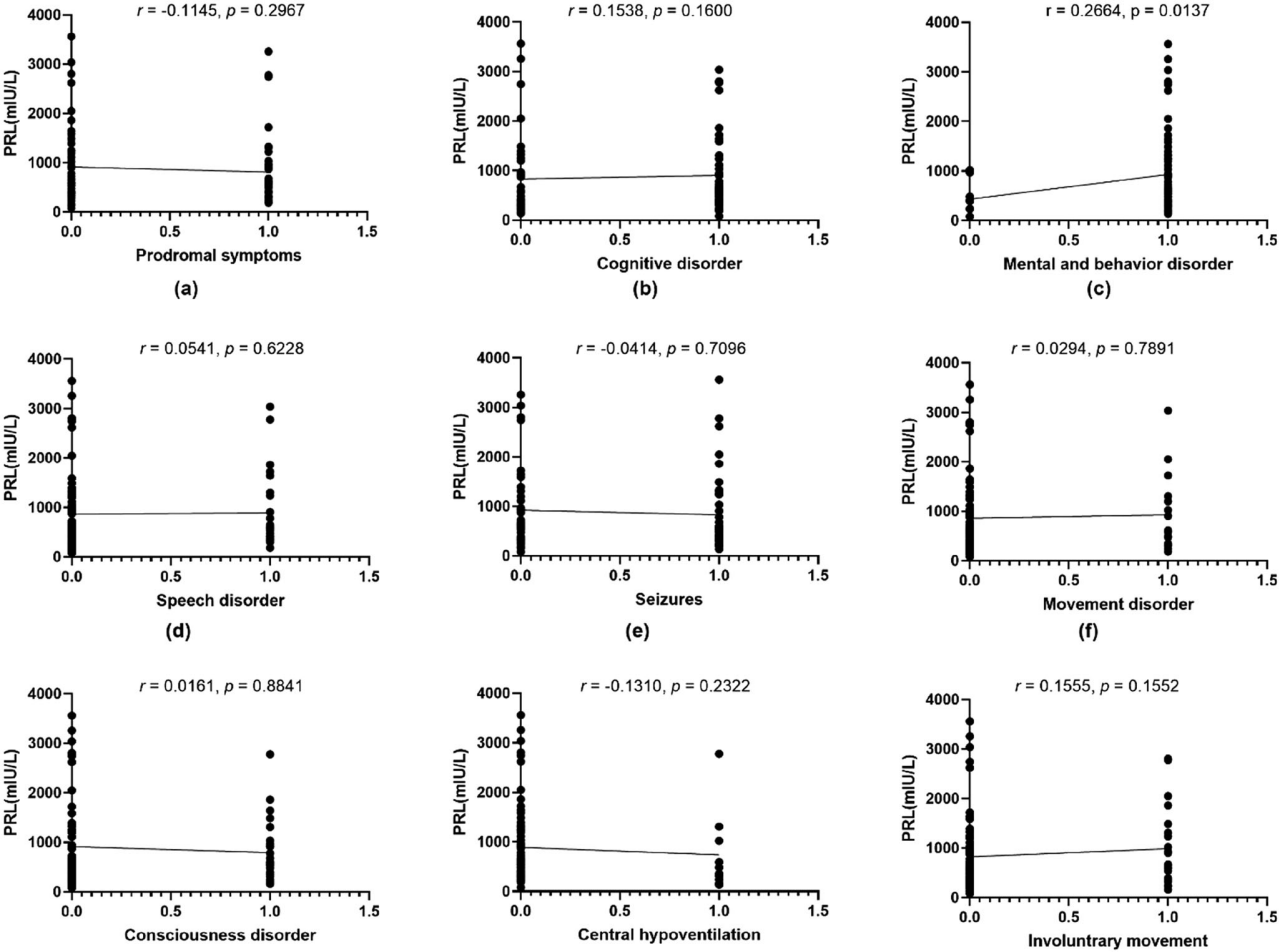
Correlation between PRL level and clinical manifestations.

As PRL was closely related to the severity of anti‐NMDAR encephalitis, we further evaluated the relationship between PRL and clinical characteristics in anti‐NMDAR encephalitis. As shown in Figure 3, we assessed the association between PRL levels and clinical symptoms, such as prodromal symptoms, psychobehavioral abnormalities, and epilepsy. And we found serum levels of PRL were associated with psychobehavioral abnormalities(*r* = 0.266, *p* = 0.014).

We further divided the anti‐NMDAR encephalitis patients into subgroups according to age, gender, severity of disease, length of stay, complication of tumor, ICU stay, therapeutic effect, and with or without abnormal brain MRI. We then compared the levels of PRL among these subgroups. As summarized in Table 2, patients with female gender, poor functional status (mRS scores > 3) had significantly higher levels of PRL than patients with male gender, good functional status (mRS scores ≤ 3) (*p* = 0.015, *p* = 0.002, respectively). There was no significant difference in other factors (Table 2).

**TABLE 2:** The levels of PRL in patients with anti‐NMDAR encephalitis.

Characteristics	PRL (mIU/L)	*p* value
**Age (years)**		
< 18 (*n* = 22)	609.05 (338.08)	0.196
≥ 18 (*n* = 63)	963.20((856.56)	
**Gender**		
Male (*n* = 24)	597.48 (511.60)	0.015
Female (*n* = 55)	1006.16 (842.32)	
**Severity of disease (mRS)**		
mRS ≤ 3 (*n* = 30)	634.33 (619.02)	0.002
mRS > 3 (*n* = 55)	1000.92 (818.92)	
**Hospital stay**		
≤ 30 days (*n* = 48)	995.63 (851.14)	0.101
> 30 days (*n* = 35)	710.55 (627.78)	
**Tumor**		
With (*n* = 24)	1065.36 (1010.35)	0.598
Without (*n* = 61)	795.28 (648.07)	
**ICU support**		
With (*n* = 30)	679.83 (590.25)	0.067
Without (*n* = 553)	976.10 (840.28)	
**Response to therapy**		
Favorable (*n* = 61)	813.59 (697.47)	0.519
Limited (*n* = 24)	1018.82 (933.02)	
**Brain MRI**		
Abnormal (*n* = 38)	787.49 (719.25)	0.353
Normal (*n* = 47)	939.49 (811.71)	

Abbreviations: Anti‐NMDAR, anti‐N‐methyl‐D‐aspartate receptor; mRS, modified Rankin Scale; PRL, prolactin; SD, standard deviation.

### 3.4 Association Between PRL Levels and Other Inflammatory Indicators in Patients With Anti‐NMDAR Encephalitis

We categorized patients with anti‐NMDAR encephalitis into Normal PRL and High PRL groups based on whether serum PRL levels fell within the normal reference range (defined as < 557.1 mIU/L for females and < 407.4 mIU/L for males). Their inflammatory statuses were compared as shown in Table 3. No statistically significant differences were observed between subgroups regarding age, gender, erythrocyte sedimentation rate (ESR), C‐reactive protein (CRP), WBC count, monocyte count in routine blood tests, or CSF parameters. However, the High PRL group exhibited significantly lower lymphocyte counts, higher neutrophil counts, and elevated NLR (*p*_adjusted_ = 0.004, *p*_adjusted_ = 0.022, and *p*_adjusted_ = 0.002, respectively).

**TABLE 3:** Lab Inflammatory results of anti‐NMDAR encephalitis patients according to PRL level.

Variables	Group 1 (*n* = 30) Normal prolactin	Group 2 (*n* = 55) High prolactin	Unadjusted *p* value	Adjusted *p* value*
PRL (mIU/L, mean (SD))	303.01 (109.97)	1181.64 (800.44)	< 0.001	< 0.001
Age (years, median [Q1,Q3])	23.0 [17.0,29.5]	22.0 [18.0,28.0]	0.800	—
Gender			0.765	—
Male	11 (36.7%)	17 (30.9%)		
Female	19 (63.3%)	38 (69.1%)		
mRS	3.00 [2.00,4.00]	4.00 [3.50,4.50]	0.002	0.003
**Blood routine**				
WBC (×109, mean (SD))	9.59 (3.03)	11.37 (4.73)	0.227	0.060
Monocytes (×109, mean (SD))	0.78 (0.39)	0.76 (0.41)	0.898	0.803
Neutrophils (×109, mean (SD))	6.76 (2.60)	8.89 (4.69)	0.130	0.022
Lymphocytes (×109, mean (SD))	1.92 (0.73)	1.47 (0.63)	0.006	0.004
NLR (median [Q1,Q3])	3.72 [2.48,5.07]	6.06 [3.54,11.4]	0.001	0.002
CRP (median [Q1,Q3])	1.30 [0.62,3.15]	2.80 [1.00,9.90]	0.042	0.156
ESR (median [Q1,Q3])	9.00 [6.25,23.2]	16.0 [10.0,24.5]	0.062	0.282
**CSF routine**				
CSF WBC (×106, median [Q1,Q3])	7.50 [2.00,25.0]	16.0 [4.00,40.0]	0.184	0.675
CSF TP (g/L, median [Q1,Q3])	0.26 [0.20,0.43]	0.24 [0.18,0.38]	0.591	0.784

Abbreviations: CRP, C‐reactive protein; ESR, erythrocyte sedimentation rate; NLR, neutrophils/lymphocyte ratio; PRL, prolactin; TP, total protein; WBC, white blood cells. *After adjusting for the effects of age and gender.

In subsequent analyses (Table 4), Spearman's rank correlation revealed significant associations between PRL levels and gender (*r* = 0.265, *p* = 0.014) as well as NLR (*r* = 0.308, *p* = 0.004). No significant correlations were identified between PRL levels and other inflammatory markers.

**TABLE 4:** Correlation analysis of possible factors that may affect PRL levels in patients with anti‐NMDAR encephalitis.

Variables	*r*	*p*
Age	0.020	0.857
Gender	0.265	0.014
**Blood routine**		
WBC	0.162	0.140
Monocytes	0.028	0.797
Neutrophils	0.149	0.172
Lymphocytes	−0.179	0.100
NLR	0.308	0.004
CRP	0.180	0.099
ESR	0.183	0.094
**CSF routine**		
CSF WBC	0.049	0.656
CSF TP	−0.084	0.447

Abbreviations: CRP, C‐reactive protein; ESR, erythrocyte sedimentation rate; NLR, neutrophils/lymphocyte ratio; PRL, prolactin; TP, total protein; WBC, white blood cells.

### 3.5 PRL Levels in Patients With Anti‐NMDAR Encephalitis During Follow‐Up

**FIGURE 4:**
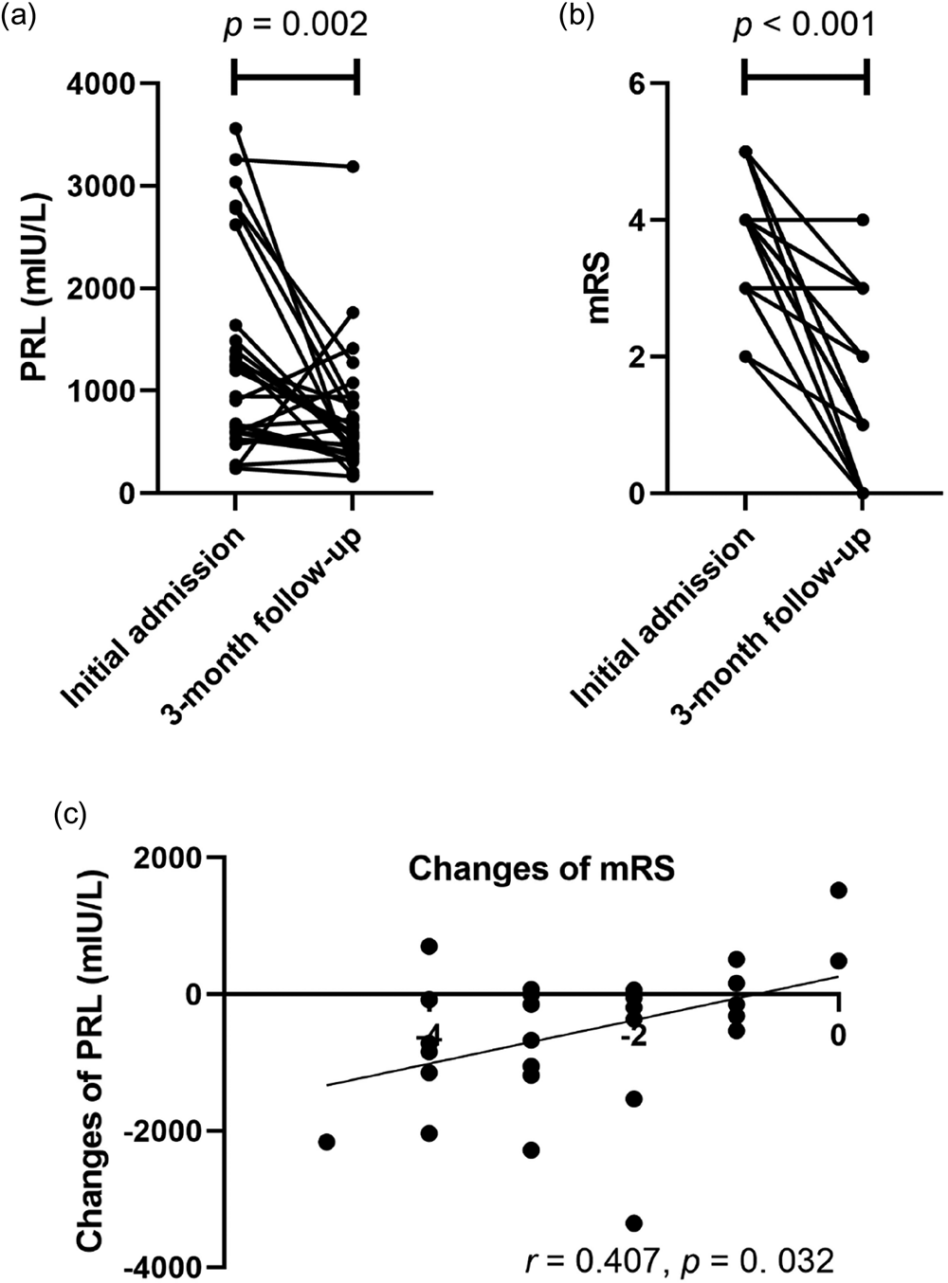
PRL levels and mRS Scores in patients with anti‐NMDAR encephalitis during follow‐up. (a) Decreased PRL levels and (b) improvement in mRS scores in anti‐NMDAR encephalitis patients between initial admission and 3‐month follow‐up after admission (*p* = 0.002, *p* < 0.001, respectively) (*n* = 28). (C) Relationship between the changes in mRS scores and the changes in the PRL levels at 3‐month follow‐up evaluation (*r* = 0.407, *p* = 0.032) (*n* = 28). mRS, modified Rankin Scale. PRL, Prolactin.

Longitudinal follow‐up of PRL levels in patients with anti‐NMDAR encephalitis revealed significant reductions in both serum PRL levels and mRS scores after 3 months of treatment (*p* = 0.002, *p*<0.001, respectively). The magnitude of reduction in PRL levels positively correlated with that of mRS scores (*r* = 0.407, *p* = 0.032) (Figure 4).

## 4 Discussion

To our knowledge, this represents the first comprehensive analysis evaluating circulating PRL levels in relation to clinical symptoms of anti‐NMDAR encephalitis. Our study revealed significantly elevated serum PRL levels in anti‐NMDAR encephalitis patients compared to HCs, with baseline PRL levels correlating with disease severity at admission. In addition, multiple studies have also indicated that an elevated NLR is associated with an inflammatory state and disease progression (Williams et al. 2014); (Ha et al. 2015). Our observation of a positive correlation between PRL and NLR aligns with reports of elevated NLR in hyperprolactinemic women, might suggesting PRL‐associated systemic inflammation (Carranza Lira et al. 2021). PRL is a multifunctional neurohormone, regulating lactation, reproduction, immune responses, and neuroendocrine functions. Among them, this study focuses on its neuroimmunomodulatory properties. PRL can activate signaling pathways such as JAK2/STAT5 and MAPK/ERK via receptor binding, influencing immune cell proliferation, differentiation, and survival (Ramos‐Martinez et al. 2021); (Borba et al. 2018). It enhances T‐cell activation (Nguyen et al. 2021) and stimulates pro‐inflammatory cytokines (e.g., TNF‐α, IL‐1β, IL‐6, IL‐12), which are pivotal in neuroinflammatory disorders like MS and experimental autoimmune encephalomyelitis (EAE) (Cejkova et al. 2009). Previous studies were consistent with our results, showing significant differences between patients with anti‐NMDAR encephalitis and HCs in terms of increased neutrophil count and decreased lymphocyte count (Zeng et al. 2019). Moreover, the study found that NLR has prognostic value in AE (Zeng et al. 2019), which further indicates that PRL may have a pro‐inflammatory effect in anti‐NMDAR encephalitis. During the onset of AE, autoantibodies (such as anti‐NMDAR antibodies) and immune cells (particularly lymphocytes) are activated, and their target is the neuronal antigens in the brain. The reason for the decrease in peripheral blood lymphocyte count may be related to the migration and recruitment of immune cells from the blood to the CNS (Dalmau et al. 2019). Other possible reasons include the increase in neutrophils and the apoptosis of some lymphocytes under a strong immune activation state, which together contribute to the elevation of NLR.

PRL's dual effects in neuroimmunological diseases are context‐dependent. In MS, characterized by immune dysregulation and CNS demyelination, PRL elevation coincides with peripheral and central immune activation (Ramos‐Martinez et al. 2021). In EAE models, PRL exacerbates acute neuroinflammation by promoting Th1/Th17 differentiation, while bromocriptine (a PRL inhibitor) ameliorates disease severity (Costanza and Pedotti 2016). Paradoxically, PRL levels decline in chronic EAE phases despite increased CSF concentrations, and PRL blockade improves the late‐onset of EAE (Zhang et al. 2019). Similarly, PRL prolongs autoreactive B‐cell survival in SLE, enhancing pathogenic autoantibody production (Y. Liu et al. 2019). PRL's ability to cross the BBB (Torner 2016) and its widespread CNS distribution—including the hypothalamus, hippocampus, and glial cells (Irving and Harvey 2014)—underscore its neuroimmune regulatory potential. These results indicate that there is a bidirectional interaction between PRL and neuroinflammation. In addition, Studies have reported that prolactin‐releasing peptide (PrRP) selectively modulates NMDA receptor‐mediated synaptic transmission in RTN neurons via postsynaptic GPR10 receptors (Xia and Arai 2011). Conversely, both systemic and intracerebroventricular administration of glutamate receptor agonists have been shown to induce a significant increase in PRL release across various animal models. Similarly, injection of an agonist for either the N‐methyl‐D‐aspartate (NMDA) receptor or kainate into the third ventricle stimulates PRL release in both cycling and lactating animals (Abbud and Smith 1993). Furthermore, administration of sodium‐D‐aspartate, a precursor of NMDA, also leads to a dose‐ and time‐dependent rise in serum PRL levels. In contrast, noncompetitive antagonists of NMDA receptors reduce plasma PRL concentrations in female rats and attenuate the preovulatory PRL surge (Wagner et al. 1993).

Notably, PRL levels in our cohort correlated with psychiatric symptoms but not seizures, prodromal fever, or sleep disturbances. This aligns with preclinical and clinical evidence linking PRL to anxiety‐like behaviors in rodents (Landgraf et al. 1999) and heightened anxiety/aggression in hyperprolactinemic women (Reavley et al. 1997). The symptoms of mental behavioral abnormalities are diverse, including mania, aggression, irritability, hallucinations, delusions, strange behaviors or depression, etc. However, in the diagnostic criteria (Graus et al. 2016) proposed by Dalmau's team in 2016 does not provide a detailed description of the specific contents of mental behavioral abnormalities. Furthermore, an estimated incidence of autoimmune encephalitides is 1.5 per million population per year (Alexopoulos and Dalakas 2019). Our article includes 85 patients, and the sample size is relatively sufficient. However, when it comes to analyzing specific mental symptoms, the sample size is relatively insufficient. Therefore, we used binary classification to assess the relationship between PRL and mental behavioral abnormalities. Hormonal fluctuations, particularly in PRL, may also contribute to stress‐related conditions, migraine headaches, sleep disturbances, and autonomic dysfunction (Salinas‐Abarca et al. 2025). Females exhibit stronger acute stress responses than males, with stress‐induced PRL elevation sensitizing nociceptors in both rodents and humans (Salinas‐Abarca et al. 2025). PrRP is primarily expressed in the noradrenergic A1 and A2 cell groups of the medulla oblongata, where it co‐localizes with noradrenaline (Fujii et al. 1999). It conveys stress‐related signals from the brainstem to hypothalamic regions, including the paraventricular nucleus (PVN)—a key center of the hypothalamic–pituitary–adrenocortical (HPA) axis (Pacak et al. 1995). Chronic stress protocols, such as repeated restraint, have been shown to elevate PrRP mRNA expression in the brainstem and shift the PrRP/tyrosine hydroxylase (TH) ratio toward PrRP (Tóth et al. 2008). Conversely, intracerebroventricular administration of PrRP was found to activate the HPA axis, resulting in increased plasma levels of adrenocorticotropic hormone (ACTH) and corticosterone through actions mediated by the PVN (Mera et al. 2006); (Samson et al. 2000). Together, these findings suggest that PRL release occurs secondary to stress yet also contributes reciprocally to the modulation of stress responses. Therapeutically, PRL‐targeting strategies, such as bromocriptine or PRL‐targeting antibodies, show promise in mitigating neuroinflammation and stress‐related pain (Stratton et al. 2025), warranting exploration in AE.

PRL's dichotomous roles—pro‐inflammatory in RA, SLE, and MS versus neuroprotective in neurodegenerative contexts—highlight its mechanistic complexity. Our longitudinal data demonstrated parallel declines in PRL and mRS scores after 3 months of treatment, supporting PRL's utility as a dynamic biomarker of disease activity. However, in our subgroup analysis, no significant PRL differences were observed between anti‐NMDAR encephalitis patients with or without teratomas/ovarian cysts, despite evidence implicating ovarian PRL in tumor invasiveness (Alkharusi et al. 2021); (Hathaway et al. 2021). Therefore, the role of PRL in the subgroups with and without tumors remains unknown. Further experimental verification is necessary to determine whether the increase in PRL in anti‐NMDAR encephalitis plays a pro‐inflammatory role or has a neuroprotective effect, especially in patients with combined tumors.

Emerging evidence challenges PRL's traditionally perceived detrimental role in neuroinflammation. Recent studies highlight its neuroprotective and pro‐myelinating properties: PRL enhances hippocampal neurogenesis (Walker et al. 2012); (Pathipati et al. 2011), improves cognition (Nguyen et al. 2021), and synergizes with interferon‐β in MS therapy (Zhornitsky et al. 2015). In spinal muscular atrophy (SMA) models, PRL improves motor function and survival (Farooq et al. 2011), while in kainate‐induced epilepsy, PRL reduces seizure severity (Tejadilla et al. 2010). Such findings suggest PRL's elevation in anti‐NMDAR encephalitis might reflect a compensatory mechanism to mitigate neuronal or glial damage.

This study has several limitations. First, the sample size, particularly the follow‐up cohort, was relatively small. Second, although age and sex differences were statistically adjusted, the inclusion of both pediatric and adult patients, stress and medication use may introduce confounding. Third, our analysis focused solely on anti‐NMDAR encephalitis; PRL dynamics in other AE subtypes remain unexplored.

## 5 Conclusions

The study demonstrated that patients with anti‐NMDAR encephalitis exhibited significantly elevated levels of circulating PRL, compared to HCs. This finding suggests that serum PRL levels may serve as a biomarker for disease severity. Notably, elevated PRL levels were also associated with high psychiatric disturbances, further underscoring its clinical relevance. PRL may emerge as a dual‐edged modulator in anti‐NMDAR encephalitis, its therapeutic targeting requires careful consideration of context‐dependent effects. Future studies should delineate PRL's stage‐specific contributions and evaluate PRL‐modulating therapies in larger, stratified cohorts.

## Author Contributions

X.M. and W.Q. contributed to the conception of the study. X.M. and W.S. collected the data and drafted the manuscript. Y.L. and W.W. helped with the data analyses. S.G., Y.S., and P.W. helped to revise and improve the manuscript. Y.S. and W.Q. polished the article. All authors agree to be accountable for the content of the work.

## Conflicts of Interest

The authors declare no conflicts of interest.

## Peer Review

The peer review history for this article is available at https://publons.com/publon/10.1002/brb3.70960

## Supporting information




**Supplementary Figures**: brb370960‐sup‐0001‐Figures.docx

## Data Availability

The data that support the findings of this study are available from the corresponding author upon reasonable request.

## References

[brb370960-bib-0001] Abbud, R. , and M. S. Smith . 1993. “Altered Luteinizing Hormone and Prolactin Responses to Excitatory Amino Acids During Lactation.” Neuroendocrinology 58: 454–464. 8284030 10.1159/000126576

[brb370960-bib-0002] Alexopoulos, H. , and M. C. Dalakas . 2019. “The Immunobiology of Autoimmune Encephalitides.” Journal of Autoimmunity 104: 102339. 31611142 10.1016/j.jaut.2019.102339

[brb370960-bib-0003] Alkharusi, A. , A. AlMuslahi , N. AlBalushi , et al. 2021. “Connections Between Prolactin and Ovarian Cancer.” PLoS ONE 16: e0255701. 34358244 10.1371/journal.pone.0255701 PMC8345882

[brb370960-bib-0004] Borba, V. V. , G. Zandman‐Goddard , and Y. Shoenfeld . 2018. “Prolactin and Autoimmunity.” Frontiers in Immunology 9: 73. 29483903 10.3389/fimmu.2018.00073 PMC5816039

[brb370960-bib-0005] Carranza Lira, S. , G. Spínola Quezada , A. L. Baez López , and E. López Muñoz . 2021. “Correlation of Prolactin Levels With the Neutrophil/Lymphocyte Ratio.” International Journal of Gynecology & Obstetrics 156: ijgo13996. 10.1002/ijgo.13996 34687230

[brb370960-bib-0006] Cejkova, P. , M. Fojtikova , and M. Cerna . 2009. “Immunomodulatory Role of Prolactin in Diabetes Development.” Autoimmunity Reviews 9: 23–27. 19248843 10.1016/j.autrev.2009.02.031

[brb370960-bib-0007] Costanza, M. , and R. Pedotti . 2016. “Prolactin: Friend or Foe in Central Nervous System Autoimmune Inflammation?” International Journal of Molecular Sciences 17: 2026. 27918427 10.3390/ijms17122026 PMC5187826

[brb370960-bib-0008] Dalmau, J. , T. Armangué , J. Planagumà , et al. 2019. “An Update on Anti‐NMDA Receptor Encephalitis for Neurologists and Psychiatrists: Mechanisms and Models.” Lancet Neurology 18: 1045–1057. 31326280 10.1016/S1474-4422(19)30244-3

[brb370960-bib-0009] Duc Nguyen, H. , N. M. H. Hoang , M. Ko , et al. 2021. “Association Between Serum Prolactin Levels and Neurodegenerative Diseases: Systematic Review and Meta‐Analysis.” Neuroimmunomodulation 29: 85–96. 34670217 10.1159/000519552

[brb370960-bib-0010] Farooq, F. , F. A. Molina , J. Hadwen , et al. 2011. “Prolactin Increases SMN Expression and Survival in a Mouse Model of Severe Spinal Muscular Atrophy via the STAT5 Pathway.” Journal of Clinical Investigation 121: 3042–3050. 21785216 10.1172/JCI46276 PMC3148738

[brb370960-bib-0011] Fujii, R. , S. Fukusumi , M. Hosoya , et al. 1999. “Tissue Distribution of Prolactin‐Releasing Peptide (PrRP) and Its Receptor.” Regulatory Peptides 83: 1–10. 10498338 10.1016/s0167-0115(99)00028-2

[brb370960-bib-0012] Graus, F. , M. J. Titulaer , R. Balu , et al. 2016. “A Clinical Approach to Diagnosis of Autoimmune Encephalitis.” Lancet Neurology 15: 391–404. 26906964 10.1016/S1474-4422(15)00401-9 PMC5066574

[brb370960-bib-0013] Ha, K.‐S. , J. Lee , G. Y. Jang , et al. 2015. “Value of Neutrophil‐Lymphocyte Ratio in Predicting Outcomes in Kawasaki Disease.” American Journal of Cardiology 116: 301–306. 25975725 10.1016/j.amjcard.2015.04.021

[brb370960-bib-0014] Hathaway, C. A. , M. S. Rice , M. K. Townsend , et al. 2021. “Prolactin and Risk of Epithelial Ovarian Cancer.” Cancer Epidemiology, Biomarkers & Prevention 30: 1652–1659. 10.1158/1055-9965.EPI-21-0139 PMC8419083 34244157

[brb370960-bib-0015] Irving, A. J. , and J. Harvey . 2014. “Leptin Regulation of Hippocampal Synaptic Function in Health and Disease.” Philosophical Transactions of the Royal Society B 369: 20130155. 10.1098/rstb.2013.0155 PMC3843886 24298156

[brb370960-bib-0016] Landgraf, R. , A. Wigger , F. Holsboer , and I. D. Neumann . 1999. “Hyper‐Reactive Hypothalamo‐Pituitary‐Adrenocortical Axis in Rats Bred for High Anxiety‐Related Behaviour.” Journal of Neuroendocrinology 11: 405–407. 10336720 10.1046/j.1365-2826.1999.00342.x

[brb370960-bib-0017] Liu, H. , X. Zhang , W. Chen , Y. Xu , X. Lin , and A. Lin . 2024. “The Relationship Between Plasma Prolactin Levels and Clinical Manifestations With Neuromyelitis Optica Spectrum Disorders.” Neurological Sciences 45: 699–707. 37620730 10.1007/s10072-023-07008-z

[brb370960-bib-0018] Liu, X. , X. Zheng , Y. Shu , et al. 2024. “Genome‐Wide Association Study Identifies IFIH1 and HLA‐DQB1*05:02 Loci Associated With Anti‐NMDAR Encephalitis.” Neurology Neuroimmunology & Neuroinflammation 11, no. 3: e200221. 10.1212/NXI.0000000000200221. 38579189 PMC11010247

[brb370960-bib-0019] Liu, Y. , Z. Zhang , Q. Jin , et al. 2019. “Hyperprolactinemia Is Associated With a High Prevalence of Serum Autoantibodies, High Levels of Inflammatory Cytokines and an Abnormal Distribution of Peripheral B‐Cell Subsets.” Endocrine 64: 648–656. 30887277 10.1007/s12020-019-01896-y

[brb370960-bib-0020] Mera, T. , H. Fujihara , M. Kawasaki , et al. 2006. “Prolactin‐releasing Peptide Is a Potent Mediator of Stress Responses in the Brain Through the Hypothalamic Paraventricular Nucleus.” Neuroscience 141: 1069–1086. 16730416 10.1016/j.neuroscience.2006.04.023

[brb370960-bib-0021] Nguyen, H. D. , B. P. Yu , N. H. M. Hoang , W. H. Jo , H. Young Chung , and M.‐S. Kim . 2022. “Prolactin and Its Altered Action in Alzheimer's Disease and Parkinson's Disease.” Neuroendocrinology 112: 427–445. 34126620 10.1159/000517798

[brb370960-bib-0022] Orbach, H. , G. Zandman‐Goddard , H. Amital , et al. 2007. “Novel Biomarkers in Autoimmune Diseases: Prolactin, Ferritin, Vitamin D, and TPA Levels in Autoimmune Diseases.” Annals of the New York Academy of Sciences 1109: 385–400. 17785327 10.1196/annals.1398.044

[brb370960-bib-0023] Pacak, K. , M. Palkovits , I. J. Kopin , and D. S. Goldstein . 1995. “Stress‐Induced Norepinephrine Release in the Hypothalamic Paraventricular Nucleus and Pituitary‐Adrenocortical and Sympathoadrenal Activity: In Vivo Microdialysis Studies.” Frontiers in Neuroendocrinology 16: 89–150. 7621982 10.1006/frne.1995.1004

[brb370960-bib-0024] Pathipati, P. , T. Gorba , A. Scheepens , V. Goffin , Y. Sun , and M. Fraser . 2011. “Growth Hormone and Prolactin Regulate Human Neural Stem Cell Regenerative Activity.” Neuroscience 190: 409–427. 21664953 10.1016/j.neuroscience.2011.05.029

[brb370960-bib-0025] Ramos‐Martinez, E. , I. Ramos‐Martínez , G. Molina‐Salinas , W. A. Zepeda‐Ruiz , and M. Cerbon . 2021. “The Role of Prolactin in Central Nervous System Inflammation.” Reviews in the Neurosciences 32: 323–340. 33661585 10.1515/revneuro-2020-0082

[brb370960-bib-0026] Reavley, S. , A. D. Fisher , D. Owen , F. H. Creed , and J. R. E. Davis . 1997. “Psychological Distress in Patients With Hyperprolactinaemia.” Clinical Endocrinology 47: 343–348. 9373457 10.1046/j.1365-2265.1997.2701073.x

[brb370960-bib-0027] Salinas‐Abarca, A. B. , M. Gamal‐Eltrabily , M. Romero‐Reyes , and S. Akerman . 2025. “The Role and Interaction of Hypothalamic‐Related Neurotransmitters in Migraine.” Journal of Headache and Pain 26: 110. 40350428 10.1186/s10194-025-02044-w PMC12067729

[brb370960-bib-0028] Samson, W. K. , Z. T. Resch , and T. C. Murphy . 2000. “A Novel Action of the Newly Described Prolactin‐Releasing Peptides: Cardiovascular Regulation.” Brain Research 858: 19–25. 10700591 10.1016/s0006-8993(99)02451-8

[brb370960-bib-0029] Stratton, H. J. , M. Dolatyari , C. Kopruszinski , et al. 2025. “A Prolactin‐Targeting Antibody to Prevent Stress‐Induced Peripheral Nociceptor Sensitization and Female Postoperative Pain.” Proceedings of the National Academy of Sciences of the United States of America 122: e2501229122. 40354542 10.1073/pnas.2501229122 PMC12107140

[brb370960-bib-0030] Tang, C. , Y. Li , X. Lin , et al. 2014. “Prolactin Increases Tumor Necrosis Factor Alpha Expression in Peripheral CD14 Monocytes of Patients With Rheumatoid Arthritis.” Cellular Immunology 290: 164–168. 24997655 10.1016/j.cellimm.2014.06.005

[brb370960-bib-0031] Tejadilla, D. , M. Cerbón , and T. Morales . 2010. “Prolactin Reduces the Damaging Effects of Excitotoxicity in the Dorsal Hippocampus of the Female Rat Independently of Ovarian Hormones.” Neuroscience 169: 1178–1185. 20570717 10.1016/j.neuroscience.2010.05.074

[brb370960-bib-0032] Torner, L 2016. “Actions of Prolactin in the Brain: From Physiological Adaptations to Stress and Neurogenesis to Psychopathology.” Front Endocrinology 7: 25. 10.3389/fendo.2016.00025 PMC4811943 27065946

[brb370960-bib-0033] Tóth, Z. E. , D. Zelena , Z. Mergl , et al. 2008. “Chronic Repeated Restraint Stress Increases Prolactin‐Releasing Peptide/Tyrosine‐Hydroxylase Ratio With Gender‐Related Differences in the Rat Brain.” Journal of Neurochemistry 104: 653–666. 18199117 10.1111/j.1471-4159.2007.05069.x

[brb370960-bib-0034] Tripathi, A. , and A. Sodhi . 2008. “Prolactin‐Induced Production of Cytokines in Macrophages In Vitro Involves JAK/STAT and JNK MAPK Pathways.” International Immunology 20: 327–336. 18187558 10.1093/intimm/dxm145

[brb370960-bib-0035] Wagner, E. J. , K. E. Moore , and K. J. Lookingland . 1993. “Sexual Differences in N‐Methyl‐D‐Aspartate Receptor‐Mediated Regulation of Tuberoinfundibular Dopaminergic Neurons in the Rat.” Brain Research 611: 139–146. 8518940 10.1016/0006-8993(93)91785-q

[brb370960-bib-0036] Walker, T. L. , J. Vukovic , M. M. Koudijs , et al. 2012. “Prolactin Stimulates Precursor Cells in the Adult Mouse Hippocampus.” PLoS ONE 7: e44371. 22973440 10.1371/journal.pone.0044371 PMC3433411

[brb370960-bib-0037] Williams, K. A. , S. I. Labidi‐Galy , K. L. Terry , et al. 2014. “Prognostic Significance and Predictors of the Neutrophil‐to‐Lymphocyte Ratio in Ovarian Cancer.” Gynecologic Oncology 132: 542–550. 24462730 10.1016/j.ygyno.2014.01.026 PMC3980949

[brb370960-bib-0038] Xia, Y.‐F. , and A. C. Arai . 2011. “Prolactin‐Releasing Peptide Enhances Synaptic Transmission in Rat Thalamus.” Neuroscience 172: 1–11. 21056089 10.1016/j.neuroscience.2010.10.079

[brb370960-bib-0039] Zeng, Z. , C. Wang , B. Wang , et al. 2019. “Prediction of Neutrophil‐to‐Lymphocyte Ratio in the Diagnosis and Progression of Autoimmune Encephalitis.” Neuroscience Letters 694: 129–135. 30521947 10.1016/j.neulet.2018.12.003

[brb370960-bib-0040] Zhang, C. , B. J. E. Raveney , H. Hohjoh , C. Tomi , S. Oki , and T. Yamamura . 2019. “Extrapituitary Prolactin Promotes Generation of Eomes‐Positive Helper T Cells Mediating Neuroinflammation.” PNAS 116: 21131–21139. 31570595 10.1073/pnas.1906438116 PMC6800326

[brb370960-bib-0041] Zhornitsky, S. , T. A. Johnson , L. M. Metz , S. Weiss , and V. W. Yong . 2015. “Prolactin in Combination With Interferon‐β Reduces Disease Severity in an Animal Model of Multiple Sclerosis.” Journal of Neuroinflammation 12: 55. 25889599 10.1186/s12974-015-0278-8 PMC4367923

[brb370960-bib-0042] Zverova, M. , E. Kitzlerova , Z. Fisar , et al. 2018. “Interplay Between the APOE Genotype and Possible Plasma Biomarkers in Alzheimer's Disease.” Current Alzheimer Research 15: 938–950. 29852871 10.2174/1567205015666180601090533

