# Supplementary material for: Elevated Prolactin Levels Are Associated With Increased Severity and Psychiatric Symptoms in Anti‐N‐Methyl‐D‐Aspartate Receptor Encephalitis
Source: Brain Behav. 2025 Oct 29;15(11):e70960. doi: 10.1002/brb3.70960 (PMC12571974; doi:10.1002/brb3.70960)
Supplement: Supplementary file 1 — Supplementary Figures: brb370960‐sup‐0001‐Figures.docx [file BRB3-15-e70960-s001.docx]

Elevated prolactin levels are associated with increased severity and psychiatric symptoms in anti-N-methyl-D-aspartate receptor encephalitis

Xiaoyu Ma^1^, Yaxin Lu^2^, Yingying Xu^1^, Shougang Guo^3^, Weiqi Wang^3^, Wei Shang^1^, Wei Qiu^4^, Pin Wang^1^*，Yaqing Shu^4, *^, Yuge Wang^4, *^

^1^ Department of Neurology, The Second Qilu Hospital of Shandong University, Jinan, China

^2^ Department of Clinical Data Center, The Third Affiliated Hospital of Sun Yat-sen University, Guangzhou, China

^3^ Department of Neurology, Shandong Provincial Hospital, Shandong First Medical University, Jinan, China

^4^ Department of Neurology, The Third Affiliated Hospital of Sun Yat-sen University, Guangzhou, China

*** Correspondence:**

Pin Wang.

Department of Neurology, The Second Qilu Hospital of Shandong University, Jinan, 250033, China

E-mail: [wangpin1023@126.com](mailto:wangpin1023@126.com)

Yaqing Shu.

Department of Neurology, The Third Affiliated Hospital of Sun Yat-sen University, Guangzhou, 510630, China.

E-mail: [shuyaq@mail.sysu.edu.cn](mailto:shuyaq@mail.sysu.edu.cn)

Yuge Wang.

Department of Neurology, The Third Affiliated Hospital of Sun Yat-sen University, Guangzhou, 510630, China.

E-mail: [wangyuge@mail.sysu.edu.cn](mailto:wangyuge@mail.sysu.edu.cn)

**Keywords: Anti-N-methyl-D-aspartate receptor encephalitis; prolactin**

**Subheading:** Prolactin in anti-NMDAR encephalitis

(a) **
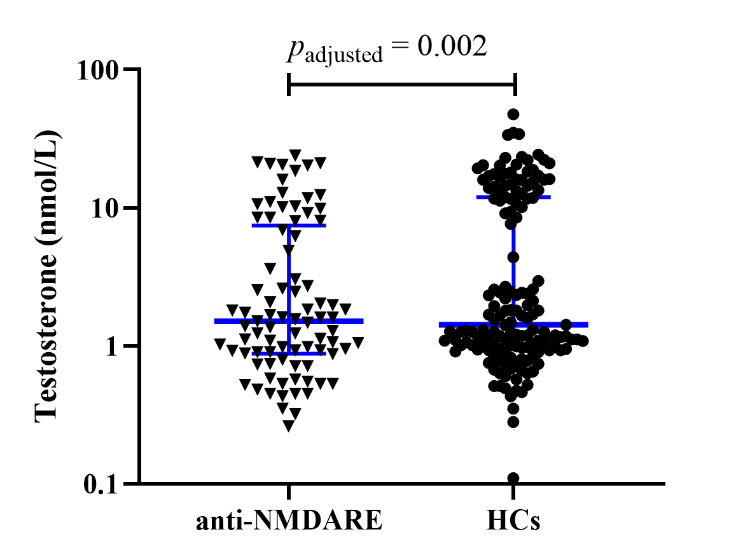
** (b)
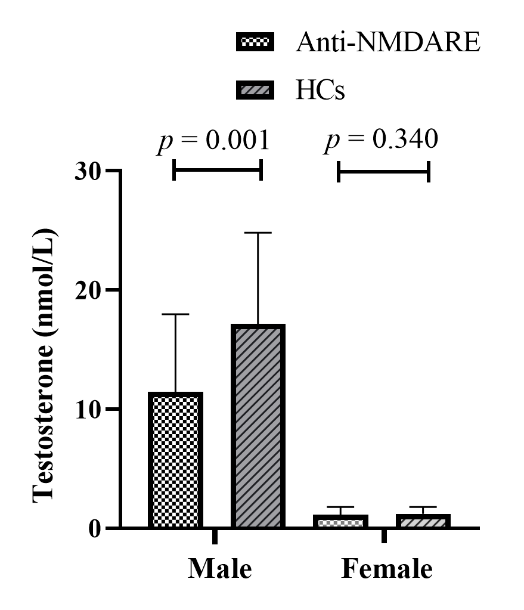


**Supplementary Figure 1. The testosterone levels in anti-NMDAR encephalitis compared with HCs. (a)**Compared with healthy controls (CTLs, n=152), testosterone levels were significantly lower in anti-NMDAR encephalitis (anti-NMDARE, n=85) at initial admission (*p* <0.184, *p*_adjusted_ =0.002). **(b)** testosterone levels were significantly lower in males with anti-NMDAR encephalitis (n=28) compared with male healthy controls (n=50, *p* = 0.001), but the difference was not significantly in females (*p* =0.340).

**(a)
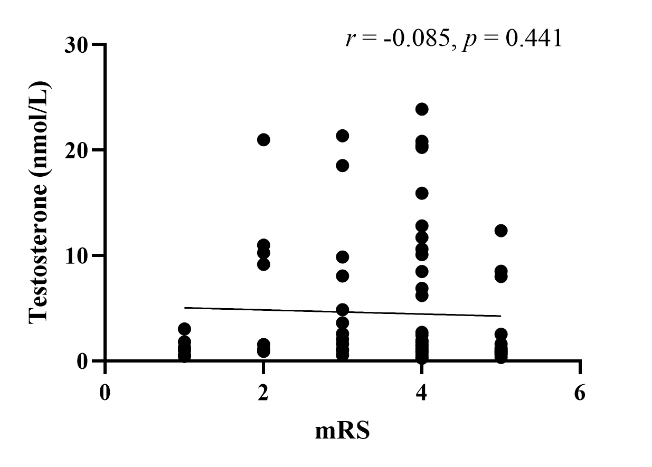
(b)**  **
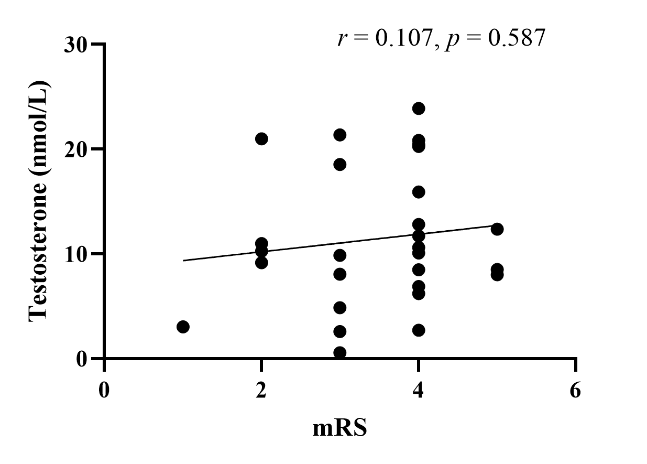
**

(c)  **
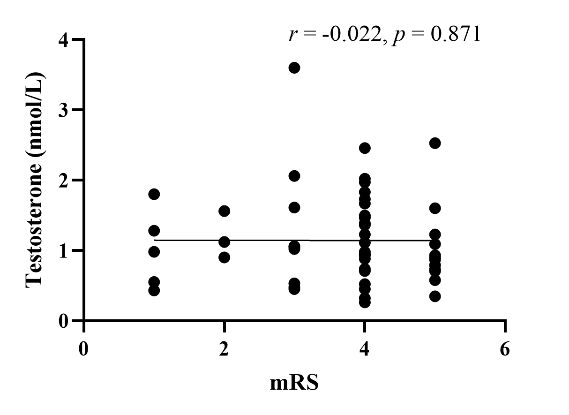
**

**Supplementary Figure 2. Relationship between testosterone and disease severity.** (a) in all patients. (b) in male. (c) in female.
